# Flavonoids from Brazilian Cerrado: Biosynthesis, Chemical and Biological Profile

**DOI:** 10.3390/molecules24162891

**Published:** 2019-08-09

**Authors:** Josana de Castro Peixoto, Bruno Junior Neves, Flávia Gonçalves Vasconcelos, Hamilton Barbosa Napolitano, Maria Gonçalves da Silva Barbalho, Sandro Dutra e Silva, Lucimar Pinheiro Rosseto

**Affiliations:** 1 Laboratório de Pesquisa em Biodiversidade (LaPeBio), Centro Universitário de Anápolis–UniEVANGÉLICA, Anápolis 75083-515, Goiás, Brazil; 2 Câmpus de Ciências Exatas e Tecnológicas, Universidade Estadual de Goiás, Anápolis 75132-400, Goiás, Brazil; 3 Laboratory of Cheminformatics (LabChemIn), Centro Universitário de Anápolis–UniEVANGÉLICA, Anápolis 75083-515, Goiás, Brazil; 4 Laboratório de Novos Materiais, Centro Universitário de Anápolis–UniEVANGÉLICA, Anápolis 75083-515, Goiás, Brazil; 5 Laboratório de Pesquisas Avançadas e Geoprocesamento (LaPAGeo), Centro Universitário de Anápolis–UniEVANGÉLICA, Anápolis 75083-515, Goiás, Brazil; 6 Laboratório de História Ambiental do Cerrado, Centro Universitário de Anápolis–UniEVANGÉLICA, Anápolis 75083-515, Goiás, Brazil

**Keywords:** biodiversity, Brazilian Cerrado, biosynthesis, flavonoids

## Abstract

Flavonoids are highly bioactive compounds with very low toxicity, which makes them attractive starting points in drug discovery. This study aims to provide information on plant species containing flavonoids, which are found in the Brazilian Cerrado. First, we present the characterization and plant diversity with emphasis on the families of flavonoid-producing plants, and then we describe the phenylpropanoid pathway which represents the flavonoids’ main route biosynthesis—generally conserved in all species. Chemical structures and biological activities of flavonoids isolated from the Cerrado’s plant species are also described based on examples from the relevant literature studies. Finally, research on the biodiversity of the Cerrado biome should be encouraged, due to the discovery of new sources of flavonoids which can provide several benefits to human health and the possibility of developing new drugs by the pharmaceutical industry.

## 1. Introduction

The Brazilian Cerrado is considered a source of bioactive substances, containing several chemical compounds with biological activities, though the loss of natural vegetation in this biome has occurred constantly as the agricultural frontiers have continued to expand. The high pressure of land use, represented by soybean or sugar cane monocultures, extensive and mechanized production of grains for export, is causing heavy loss of vegetation. It is estimated that 31% to 34% of the remaining regions of the Cerrado could disappear by 2050, thus it is considered one of the 25 hotspots worldwide for the conservation of biodiversity [1,2]. In this sense, the propagation of knowledge related to the importance of the plant species found in the Cerrado could contribute to the preservation of this biome.

In dry forests such as those observed in the Cerrado, plant species have many polyphenolic compounds, such as flavonoids and tannins. Flavonoids are the main class of polyphenols, and it is estimated to comprise over 8000 metabolites [3], which are very common in sessile organisms probably because of exposure to water stress, intense sunlight, herbivorous attacks, and fungal infections [4]. For example, it is suggested that vicenin-2 flavonoid, isolated in leaves from Brazilian wild *Lychnophora* plants, acts as a UV light barrier to protect the plant from adverse external conditions.

The wide variety and distribution of flavonoids, together with their relatively low toxicity compared to other active plant compounds (for instance alkaloids), enable humans to ingest a significant amount of flavonoids. Furthermore, their consumption has been suggested to present a wide range of health benefits [3]. In order to compile information about the Cerrado’s flavonoids and to highlight their pharmacological potential, a review of the literature available, from the characterization of this biome to its chemical and biological diversity, is presented.

## 2. Characterization and Plant Diversity of the Brazilian Cerrado

**Figure 1:**
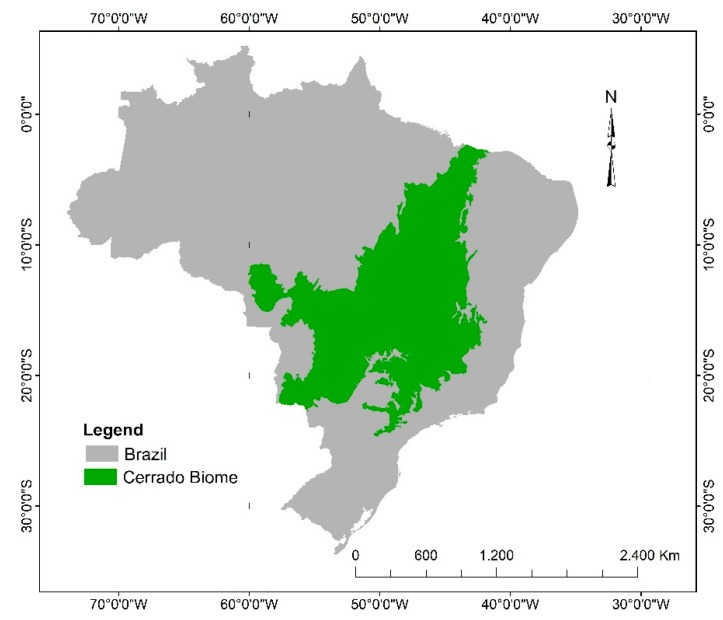
Brazil map highlighting the Cerrado biome [6]. Political Limit–Ministry of the Environment. Elaboration and Organization: Maria Gonçalves da Silva Barbalho LAPAGeo/PPSTMA/UniEVANGÉLICA.

Brazil has one of the largest genetic diversities in the world, enclosed in six main biomes: Amazon Forest, Caatinga, Pantanal, Atlantic Forest, Campos do Sul and Cerrado. Among the world’s savannas, the Cerrado biome is considered to possess the greatest plant diversity, and most of its extension is located in the Central Plateau of Brazil, including, among other parts of the federation, the state of Goiás (Figure 1) [5,6]. The presentation of the Brazilian Cerrado comes from its importance in the classification in relation to the other national biomes. It is considered the second largest biome in South America; it has being surpassed only by the Amazon and comprises about 24% of the national territory and part of Paraguay and Bolivia [7].

The Cerrado is considered a critical area for the conservation of biodiversity in the world (hotspots), as a consequence of anthropic actions [8]. Its original area surpasses two million km^2^ and is characterized by being a set of vegetal formations that presents physiognomy and variable floristic compositions: country, savanna, and forest, forming an ecological complex mosaic [9].

In the vegetation complex which composes the Cerrado biome there is a great variety of ecological systems, types of soils, climate, relief and altitude [10]. The peculiar combination of edaphic and climatic conditions in almost all its extension results in characteristic vegetation: *xeromorfa*, and in a seasonal climate (approximately 6 dry months) [11]. The Cerrado’s vegetation is in a condition of high vulnerability considering advances in the agricultural frontier initiated in the 1970s from governmental incentives for occupation of the Cerrado and adoption of mechanization, entailing serious threats to many endemic species [5,12]. This process was driven by high anthropization and the advance of monocultures and pastures, which may have led many plant species to extinction before they had even been recorded. According to Strassburg et al. (2017), about half of the original 2 million square kilometers of the Cerrado were transformed into planted pastures, annual crops, and other types of use [2]. Pastures planted with grasses of African origin currently cover an area of 500,000 km^2^, or equivalent to the area of Spain. Monocultures are cultivated in another 100,000 km^2^, mainly soybeans. The total area for conservation is about 33,000 km^2^, clearly insufficient when compared to the main land uses in the Cerrado. In 2000, the Brazilian Cerrado was included in the list of 25 “hotspots” in the world, that is, among the important biomes of the globe characterized by a high concentration of endemic species and experiencing an exceptional loss of habitat [8]. Such areas are of paramount importance for the maintenance of global biodiversity and therefore need to be conserved.

Moreover, in the conservation context of a biome’s biodiversity, it is necessary to take into account the socio-cultural relations involved. Environmental degradation threatens not only biodiversity, but also the cultural heritage of the populations that make use of this biodiversity. For instance, the knowledge of the medicinal use of several species, which is transferred from generation to generation, and which may disappear with the extinction of the species. On the other hand, these empirical and cultural practices, when poorly conducted by the population, may lead to unsustainable extractivism. In sum, biotechnologies and the sustainability of native biodiversity need, as Rigonato (2011, p. 321) says, “Guarantee the socio-cultural equity of human beings who historically try to survive in harmony with the Cerrado” [13]. There is, consequently, a pressing need to generate knowledge and develop processes, based on a multidisciplinary effort that alters socioeconomic improvement and preservation of the species in the Cerrado.

In the Cerrado biome, over 12 thousand species of vascular plants have been cataloged, of which several have more than one regional use with a strong cultural and economic impact on local communities [5]. The most frequent and important uses of the Cerrado plant species occur in rural properties, and several species have stood out for food and medicines importance [12]. Such uses indicate the great commercial potential of Cerrado species and have aroused the attention of agroindustry and the pharmaceutical and food industries. Many species produce fruits rich in vitamins (mainly A, C, and E) [14] and antioxidants [15], which could be inserted into sustainable production systems. The evaluation of the therapeutic potential of plant species and some of their constituents, such as flavonoids, alkaloids, triterpenes, sesquiterpenes, tannins, lignans, etc., has been the subject of incessant studies where pharmacological actions have already been proven through pre-clinical trials with animals [16]. Many of these substances have great potential for future use as medicinal agents [17]

Among all classes of bioactive compounds, flavonoids (in Rutaceae, Lauraceae, and Myristicaceae) deserve close attention because they represent 13% of all specialised metabolites of the Brazilian biodiversity, according to the Nuclei of Bioassays, Ecophysiology and Biosynthesis of Natural Products Database (NuBBE_DB_) website [18]. However, these data are limited because only about 5% of all chemical information on Brazilian biodiversity is available at NuBBE_DB_.

Flavonoids are directly responsible for the therapeutic activity of plants. Their anti-inflammatory [19,20], antimicrobial [21], antioxidant [22] actions, and potential for biotechnological applications, make Cerrado plants good candidates for phytochemical studies since these compounds are found in high concentrations in many fruits, flowers and vegetables. For example, by chemical characterization and bioprospecting of cashew tree polysaccharide of the Brazilian Cerrado (*Anacardium othonianum* Rizz.), Anacardiaceae were detected galactomannan and flavonoid compounds [23].

**Figure 2:**
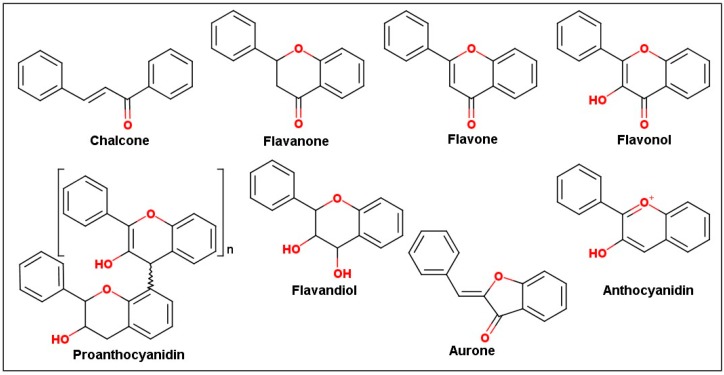
Major subclasses of flavonoid structures [3,24].

The flavonoids’ biological activities depend largely on their structural diversity. They can be subdivided into seven main subclasses: chalcones (naringenin), flavones (apigenin), flavonols (quercetin, kaempferol), flavanones (naringenin), anthocyanins (cyanidin), flavandiols (leucocyanidin), and proanthocyanidins or condensed tannins, whereas aurons are found in some species (Figure 2) [3,24].

Structural diversity in each flavonoid subclass arises from the various hydroxylation, methoxylation, glycosylation, sulphation, and acylation patterns of ring substitutions [25,26]. Glycosylation is essential for the stable accumulation of flavonoids, and makes the flavonoid less reactive and more water soluble, allowing its storage in the cell vacuole [27].

### 2.1. Biosynthesis of Flavonoids

**Figure 3:**
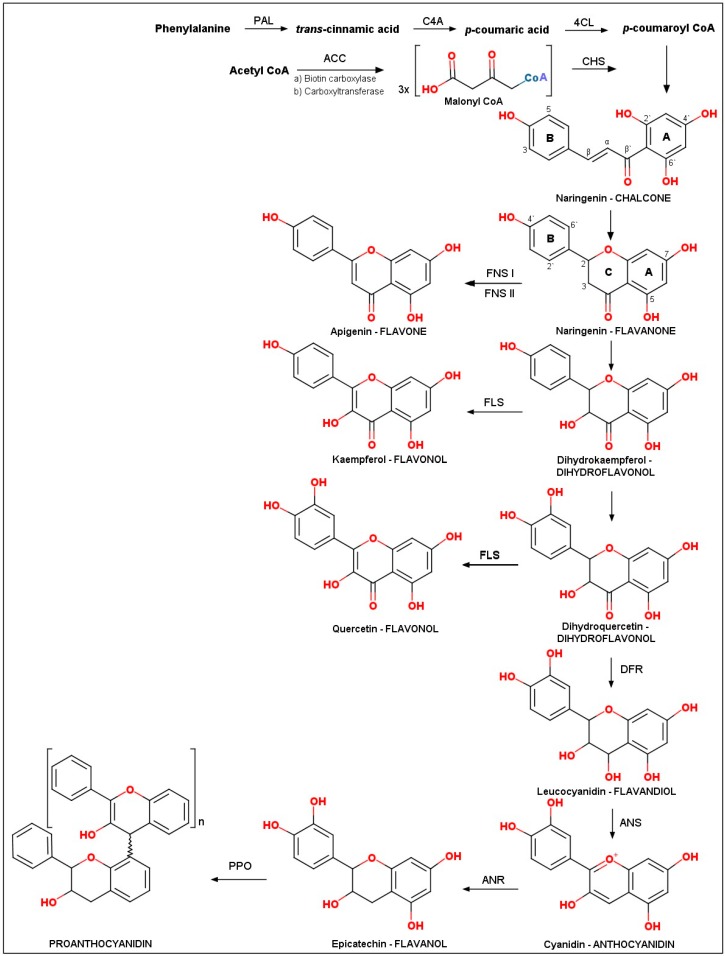
**Schematic of the major branch pathways of flavonoid biosynthesis.** Adapted from [28]. PAL, phenylalanine ammonia-lyase; C4H, cinnamic acid 4-hydroxylase; 4CL, 4-coumaric acid: CoA ligase; ACC, acetyl-CoA carboxylase; CHS, chalcone synthase; CHI, chalcone isomerase; FNS I/FNS II, flavone synthases; F3H, flavanone 3-hydroxylase; F3`H, flavonoid 30-hydroxylase; FLS, flavonol synthase; DFR, dihydroflavonol 4-reductase; ANS, anthocyanidin synthase; ANR, anthocyanidin reductase; PPO, polyphenol oxidase.

Flavonoid biosynthesis pathways vary among species [3], however, the main trunk pathway of flavonoids is described in this review, since this is generally conserved in all plant species. We emphasize the enzymes responsible for the major subclasses of flavonoids (Figure 2), but we do not discuss the genes encoding these enzymes. In this sense, genes that encode enzymes for biosynthetic reactions have been characterized by efforts that combine reverse genetics with molecular phenotyping and, therefore, we refer the reader to the reviews of Tohge et al. (2017) and Saito et al. (2013) for more details [3,28]. The flavonoid basic structure has a three-ring diphenylpropane (C6–C3–C6) core structure and the skeleton contains two benzene rings, which are connected by a C3 moiety. An oxygen-containing pyran ring forms an aliphatic chain or a six-membered ring C attached to ring A [29]. The various subclasses of flavonoids differ in their level of oxidation of the C ring of the basic 4-oxoflavonoid (2-phenyl-benzo-c-pyrone) nucleus (Figure 3).

Flavonoid synthesis occurs at the convergence of the shikimate and acetate pathways, which have as precursors molecules phenylalanine and acetyl CoA, respectively. The former provides *p*-coumaroyl-CoA and the latter is responsible for C2 chain elongation by utilizing malonyl-CoA as the condensing unit [30,31]. Phenylalanine synthesized by the shikimate pathway is cleaved by phenylammonia-lyase (PAL) to yield ammonia and *trans*-cinnamic acid. Subsequently, cinnamic acid 4-hydroxylase (C4H), a cytochrome P450 monooxygenase, catalyzes the hydroxylation of *trans*-cinnamic acid at C-4 position yielding *p*-coumaric acid. Then, *p*-coumaric acid needs to be activated by an ATP-consuming condensation reaction catalyzed by 4-coumaric acid:CoA ligase (4CL), yielding *p*-coumaroyl-CoA [32]. Malonyl-CoA formation is catalyzed by an acetyl-CoA carboxylase (ACC), an Mg^2+^ATP dependent protein. Synthesis malonyl-CoA from acetyl-CoA takes place in two steps: a) bicarbonate is transferred to the biotin molecule covalently bound to ACC (biotin carboxylase) through the hydrolysis the ATP, b) transfer of the carboxyl group to acetyl-CoA yield malonyl-CoA (carboxyltransferase) [33].

Flavonoid scaffold formation occurs by a complex series of reactions including condensations, isomerizations, oxidations, and reductions under the initial action of chalcone synthase (CHS), which belongs to a family of type III polyketide synthase [28]. CHS catalyzes the Claisen-ester condensation from three molecules of malonyl-CoA and *p*-coumaroyl-CoA, resulting in the formation of the first flavonoids, naringenin chalcone (4,2`,4`,6`-tetrahydroxychalcone). This chalcone is isomerized by a chalcone isomerase (CHI) to yield flavanone (2*S*)-naringenin from the stereospecific cyclization of naringenin chalcone [34,35].

The flavanone conversion to flavone (apigenin) has been performed by two different flavone synthases (FNS I and FNS II), a P450 enzyme in most plants [36]. (2*S*)-naringenin also is oxygenated at 3-position by flavanone 3-hydroxylase (F3H)—a 2-oxoglutarate-dependent dioxygenases (2-ODDs) yielding CO_2_, succinate and dihydrokaempferol (dihydroflavonol). This and the kaempferol are substrates to flavonoid 3`-hydroxylase (F3`H)—a cytochrome P450 monooxygenase responsible for hydroxylation of 3′-position of B-ring of these flavonoids converting them, respectively, into dihydroquercetin and quercetin.

The biosynthesis of flavonols (kaempferol and quercetin) occurs under the action of flavonol synthase (FLS). This enzyme (a 2-ODD) catalyzes the formation of the double bond between C-2 and C-3 of dihydrokaempferol and dihydroquercetin. The keto group of dihydroquercetin is reduced by dihydroflavonol reductase (DFR) yielding leucocyanidin (flavandiol). Then, the enzyme anthocyanidin synthase (ANS) catalyzes the formation of colored anthocyanidin from leucocyanidin, with 2-oxoglutarate and oxygen as co-substrates [28]. For the formation of flavanols and condensed tannins, the anthocyanidin reductase (ANR), an enzyme NADPH-dependent catalyzes the stereoselective reduction of cyanidin to form (−)-epicatechin. Oxidative polimerization of (−)-epicatechin is catalyzed by polyphenol oxidase (PPO), a laccase type enzyme.

The phenylpropanoid pathway presented is the main biosynthesis pathway for the major flavonoid subclasses, and much of the understanding of this important process comes from the work done in the *Arabidopsis thaliana* model [28]. Most of the enzymes and genes involved in flavonoids biosynthesis have been characterized, however, it is still unclear whether there are other routes [37] as new structures are discovered and as a consequence new metabolic pathways. On the other hand, the biosynthesis of metabolites is influenced by various environmental factors (light quality, UV irradiation, temperature, nutrient deficiency, and pathogen attack). Light is one of the most important environmental factors that affect the accumulation of flavonoids in plants. Pedroso et al. (2017) demonstrated that the flavonol rutin production was higher under white and blue lights for the in vitro culture of *Hyptis marrubioides* Epling, Lamiaceae, a species from the Brazilian Cerrado [38].

### 2.2. Chemical Profile of Flavonoids

The investigation of the multiple chemical structures of the flavonoids and how these structures influence their biological profile and environmental properties are essential to the Brazilian Cerrado, impacting strongly on the discovery of new drugs such as anticancer agents [39]. In order to understand how the molecular arrangement can exhibit the significant variations of the physical chemical properties of flavonoids, the crystallographic study leads to molecular structure [40]. Crystallography is a scientific methodology which enables the discovery of the structure of a flavonoid compound, as long as the compound is in a crystalline solid state [41,42,43,44].

The flavonoid molecular skeleton consists of two phenyl rings and a heterocyclic ring (Figure 3). They include chalcone compounds, which belong to a subclass of flavonoid intermediates with an important biological profile due to their presence in many pharmaceutical compounds [42,43,44,45,46,47,48,49]. Chemically, chalcone flavonoids are characterized by aromatic rings bonded through a three-carbon bridge having a keto carbonyl group and one α,β-unsaturation. The knowledge of the three-dimensional structure is important in the structural chemistry of flavonoids. The crystallographic methodology is based on the X-ray diffraction from a single-crystal and studies of the atomic arrangement in the solid state [50]. Once the structural model of a flavonoid is obtained, both the accurate atomic molecular position and the supramolecular arrangement are assigned.

**Figure 4:**
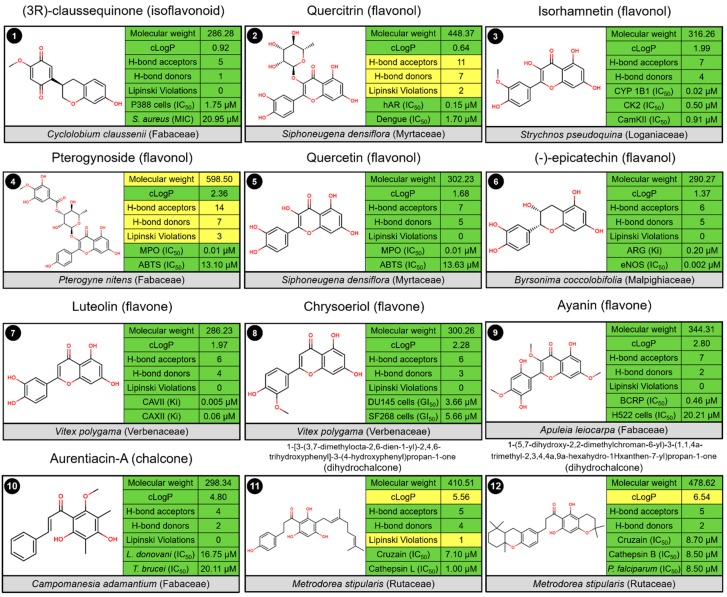
Examples of promising starting points for drug discovery.

Flavonoids, except glycosylated molecules and chalcones, have adequate physicochemical properties (i.e., cLogP, molecular weight, and number of hydrogen-bond acceptors and donors), which indicated drug-likeness potential for Cerrado’s main flavonoid subclasses [51]. By excavating the relationship between chemical structures and biological effects, results showed the core scaffold and side chain in flavonoids may lead to different biological functions (Figure 4). The carboxyl combination and olefin group make a reactive keto-ethylene chain that may promote the conjugation between the two aromatic rings. This feature holds chalcone molecules in almost flat structures which increases their biological potential [52,53,54]. This commonly observed planarity, associated with the conjugated double bonds present in chalcone skeleton structures, is capable of promoting an electronic delocalization on the aromatic rings. The structures of the naturally occurring flavonoids isolated from the Cerrado`s plant species, their natural source, are shown in Table 1.

### 2.3. Biological Profile of Isolated Flavonoid

In the following paragraphs, the most promising flavonoids isolated from the Cerrado’s plant species will be discussed based on examples from the relevant literature studies. The discussion will be focused on examples regarding the chemical properties and biological activities of pure compounds. Unfortunately, the biological profile of these Brazilian flavonoids is little explored in scientific publications. Therefore, the biological activities of these molecules were extracted from the ChEMBL database [60]. The main findings are grouped and summarized in Figure 4.

The (3*R*)-claussequinone (**1**) is the major isoflavonoid isolated from heartwood extract *Cyclolobium claussenii*, a plant of Fabaceae family popularly known as “sucupira carim” [61]. The molecule displayed a half maximal inhibitory concentration (IC_50_) of 1.75 µM against the P388 lymphocytic leukemia test system in vitro, and reasonable antibacterial activity against *Staphylococcus aureus*, with minimum inhibitory concentration (MIC) of 20.95 µM [21].

In the flavonols subclass, quercitrin (**2**), isolated from *Siphoneugena densiflora* (Myrtaceae) [19], has been reported to inhibit human aldose reductase (hALR) with an IC_50_ of 0.15 µM [62]. The hALR reduces the aldehyde form of glucose to sorbitol by using NADPH as a cofactor. It plays a key role in many of the complications arising from diabetes. Hence, quercitrin represents a promising therapeutic agent for preventing diabetic complications, such as neuropathy, nephropathy, retinopathy, and cataracts [63,64]. In addition, quercitrin has been observed to interfere with dengue virus (DENV) replication with IC_50_ of 2.1 µM [65]

Another promising flavonol is isorhamnetin (**3**). This molecule was isolated from leaves of *Strychnos pseudoquina* (Loganiaceae), a plant popularly known as “quina do campo” [66]. Takemura and co-workers (2010) showed that isorhamnetin is a potent inhibitor of cytochrome P450 (CYP) 1B1 (IC_50_ = 0.02 µM). CYP 1B1 catalyzes 17β-estradiol to 4-hydroxyestradiol. The 4-hydroxyestradiol is a risk factor for carcinogenesis since it exerts a strong agonistic effect for the estrogen receptor (ER), accelerating proliferation of estrogen-dependent cells [67]. In addition, Wang and co-workers (2009) showed that isorhamnetin inhibits mammalian casein kinase 2 (CK2, IC_50_ = 0.50 µM) and Ca^2+^/calmodulin-dependent protein kinase II (CaMKII, IC_50_ = 0.91 µM), blocking the overactivation of heat shock protein 70 (HSP70). So, isorhamnetin represents an interesting anticancer scaffold for prospective hit-to-lead investigations [39].

Two flavonols, pterogynoside (**4**) and quercetin (**5**), isolated from *Pterogyne nitens* (Fabaceae) [20] and *S. densiflora* [19], respectively, are promising anti-inflammatory agents. Both compounds exhibited potent IC_50_ values (0,01 µM) against myeloperoxidase (MPO), a heme-enzyme present in human neutrophils that plays an important role in infection and inflammation. Pterogynoside and quercetin showed higher scavenging activity towards 2,2′-azino-bis(3-ethylbenzothiazoline-6-sulphonic acid) (ABTS) radical, with values of IC_50_ around 13.30 µM [20].

In the flavanols subclass, (−)-epicatechin (**6**) isolated from the leaves and stems of *Byrsonima coccolobifolia* (Malpighiaceae) has been reported to inhibit arginase (ARG) of *Leishmania amazonensis* with a dissociation constant (Ki) of 0.20 µM [68]. ARG is associated with the production of nitric oxide (NO) molecules, high concentrations of which could kill the parasites [69]. Surprisingly, (−)-epicatechin is able to activate endothelial nitric oxide synthase (eNOS, IC_50_ = 0.002 µM) in bovine coronary artery endothelial cells and thereby increase NO production [69]. Physiological increases in NO have been linked with vasodilation of endothelial cells and are promising for the preservation of cardiovascular health [68].

Promising biological activities have also been reported for two flavones, luteolin (**7**) and chrysoeriol (**8**), isolated from fruits of *Vitex polygama* (Verbenaceae) [70]. Karioti and co-workers (2015) shown that luteolin is a potent inhibitor of human mitochondrial carbonic anhydrases (CA) isoforms VII and XII (IC_50_s = 0.005 and 0.06 µM, respectively) [71]. Moreover, luteolin showed appropriate selectivity indexes (SIs) for the inhibition of the offtarget isoforms, such as the cytosolic CA isoforms I and II (SI > 61.2) [71]. On the other hand, chrysoeriol has been reported as a potent anticancer candidate, inhibiting growth of human prostate cancer (DU145) and glioblastoma (SF268) cells with GI_50_ values of 3.66 µM and 5.66 µM, respectively [72].

Ayanin (**9**), a flavone isolated from the wood of *Apuleia leiocarpa* (Fabaceae) [73], was found to be a promising modulator of breast cancer resistance protein (BCRP) due to its high inhibitory potency (IC_50_ = 0.46 µM) [74] and low toxicity (e.g., CC_50_ of 20.22 µM against H522 cells) [75]. In this sense, the consumption of ayanin could change the pharmacokinetics and drug levels of anticancer drugs that are BCRP substrates [74].

In the chalcones subclass, aurentiacin-A (**10**), isolated from fruits of *Campomanesia adamantium* (Myrtaceae) [76], has been reported to exhibit antiprotozoal activity against *Leishmania donovani* and *Trypanosoma brucei* at low micromolar concentrations, with IC_50_s of 16.75 µM and 20.11 µM, respectively [77]. Although the compound has unsatisfactory potency to proceed with in vivo assays, it represents a promising starting point for hit-to-lead studies.

*Metrodorea stipularis* stem extracts were studied in the search for possible antichagastic, antimalarial, and antitumoral compounds. Two dihydrochalcones, 1-[3-(3,7-dimethylocta-2,6-dien-1-yl)-2,4,6-trihydroxyphenyl]-3-(4-hydroxyphenyl)propan-1-one (**11**) and 1-(5,7-dihydroxy-2,2-dimethylchroman-6-yl)-3-(1,1,4a-trimethyl-2,3,4,4a,9a-hexahydro-1H-xanthen-7-yl)propan-1-one (**12**), showed significant inhibitory activity. Compound **11** displayed IC_50_ values of 7.10 µM and 1.00 µM against cruzain of *Trypanosoma cruzi* and human cathepsin L, respectively. Compound **12** displayed significant activity against cruzain (IC_50_ = 8.70 µM) and human cathepsin B (IC_50_ = 8.50 µM). Cruzain is an enzyme that plays an essential role in *T. cruzi* by promoting digestion of proteins; thus, it is an interesting biological target in the search for new therapies for Chagas disease. Cathepsins B and L are cysteine proteases involved in progression of tumors. In addition, compound **12** showed activity (IC_50_ = 8.50 µM) against *Plasmodium falciparum* multi-drug-resistant (W2) strain [78].

## 3. Conclusions

In this review, biosynthetic aspects, chemical profiles, and biological activities have been presented, showing that despite the great natural product biodiversity in the Cerrado biome, flavonoids are little explored. It was verified that besides the antioxidant and antimicrobial properties common to flavonoids, other promising results have been reported, such as anticancer, antiprotozoan, and antichagasic activity. The daily natural product consumption of the Cerrado biome containing flavonoids could prevent chronic diseases, parasitic diseases, and premature aging, and still be used as a solution to one of the main public health problems in Brazil, dengue, due to the antiviral activity reported. The beneficial properties of flavonoids for health should stimulate the food and pharmaceutical industries to develop new products promoting the sustainable development of regions with characteristics of the Cerrado.

## Figures and Tables

**Table 1:** Flavonoids isolated from the Cerrado’s plant species, Brazil [55,56,57,58,59].

Species Name	Family	Flavonoids
*Secondatia densiflora* A.DC.	Apocynaceae	Pterogynoside; quercetin
*Tithonia diversifolia* (Hemsl)	Asteraceae	Luteolin
*Apuleia leiocarpa* (Vogel) J.F.Macbr.	Fabaceae	Ayanin
*Bauhinia candicans* Benth.	Fabaceae	Kaempferol 3-*O*-rutinoside; Kaempferol 3-*O*-rutinosidium
*Bauhinia forficata* Link.	Fabaceae	kaempferitrine
*Bauhinia guianensis* Aubl.	Fabaceae	4-hydroxy-7-methoxyflavone
*Bauhinia manca* Standl.	Fabaceae	Apigenin; Chrisoeriol; Luteolin 5,3-dimethoxy; Kaempferol; Isoliquiritigenin; 2-methoxy isoliquiritigenin; 4-methoxy isoliquiritigenin; Echinatine; 2,4-dihydroxy-4-methoxy-dihydrochalcone; (2*S*)-Narigenin (2*S*)-Eriodiethiol; (2*S*)-Lithiocygenin; (2*S*)-Lithiocygenin 7-methoxy; (2*S*)-Lithiocygenin 4-methoxy; (2*S*)-7,4-Dihydroxyflavone; (2*S*)-7,3-Dimethoxy-4-hydroxy-flavone; (2*S*)-3,4-Dimethoxy-7-hydroxy-flavone; (2*S*)-7,4-Dimethoxy-3-hydroxy-flavone
*Bauhinia megalandra Griseb.*	Fabaceae	5,7,5′-trihydroxy-2′-*O*-rhamnosyl-flavone; 5,7,2′-trihydroxy-5′-*O*-rhamnosyl flavone
*Bauhinia purpúrea* DC. ex Walp.	Fabaceae	Soquercitrin; Quercetin; Astragalin
*Bauhinia reticulata* DC.	Fabaceae	Quercetin
*Bauhinia splendens* Kunth	Fabaceae	Bausplendin, Quercetin, Routine
*Bauhinia tomentosa* Náves ex Fern.-Vill.	Fabaceae	Isoquercitrin; Quercetin; Routine
*Bauhinia vahlii* Wight & Arn.	Fabaceae	Quercetin; Quercetin-3-glucoside; Kaempferol; Agathisflavone
*Bauhinia. variegata* L.	Fabaceae	Narigenin-5,7-dimethoxy-4-rhamnoglycoside; Kaempferol-3-galactoside; Kaempferol-3-ramno-glucoside
*Cyclolobium claussenii* Benth.	Fabaceae	(3*R*)-claussequinone
*Pterogyne nitens* Tull.	Fabaceae	Pterogynoside; quercetin
*Strychnos pseudoquina* A. St. Hil.	Loganiaceae	isorhamnetin
*Byrsonima coccolobifolia* Kunth	Malpighiaceae	(−)-epicatechin
*Campomanesia adamantium* (Cambess.) O.Berg	Myrtaceae	Aurentiacin-A
*Siphoneugena densiflora* O.Berg.	Myrtaceae	Quercetin
*Metrodorea stipularis* Mart.	Myrtaceae	1-[3-(3,7-dimethylocta-2,6-dien-1-yl)-2,4,6-trihydroxyphenyl]-3-(4-hydroxyphenyl)propan-1-one 1-(5,7-dihydroxy-2,2-dimethylchroman-6-yl)-3-(1,1,4a-trimethyl-2,3,4,4a,9a-hexahydro-1H-xanthen-7-yl)propan-1-one
*Genipa americana L*	Rubiaceae	Quercetin-3-*O*-robinoside, Kaempferol-3-*O*-robinoside, Isorhamnetin-3-*O*-robinoside, Kaempferol-3-*O*-robinoside-7-*O*-rhamnoside (robinin), Isorhamnetin-3-*O*-robinoside-7-rhamnoside.
*Spiranthera odoratissima* A. St.-Hil.	Rutaceae	Kaempferol-3-ramno-glucoside
*Vitex polygama* Cham.	Verbenaceae	Luteolin; chrysoeriol
*Qualea grandiflora* Mart.	Vochysiaceae	kanferol-3-OAL-(4”-Z*p*-cumaroyl) -raminoside (2), squalene, phytol, lupeol, A-amyrin, B-amirin, sitosterol, ursolic and oleanolic acids and sitosterol 3-OBD-glucopyranoside.
